# Simultaneous G9a inhibition and histamine H3 receptor antagonism modulate autism-like behaviours and neuroinflammation in BTBR T+tf/J Mice

**DOI:** 10.1192/j.eurpsy.2025.1700

**Published:** 2025-08-26

**Authors:** M. Hajar, H. Stark, B. Sadek

**Affiliations:** 1 Pharmacology and Therapeutics, College of Medicine and Health Sciences, Al Ain, United Arab Emirates; 2 Institute of Pharmaceutical and Medicinal Chemistry, Heinrich Heine University Düsseldorf, Düsseldorf, Germany

## Abstract

**Introduction:**

Autism Spectrum Disorder (ASD) is a complex neuropsychiatric disorder characterized by deficits in social interaction, anxiety and the presence of repetitive-ritualistic behaviours. Recent studies suggest that epigenetic mechanisms, such as histone methylation, play a crucial role in the etiology of ASD by regulating gene expression linked to neuronal differentiation and proliferation. The enzyme, G9a is responsible for histone H3K9 methylation, and its inhibition has shown promise in altering epigenetic pathways associated with ASD onset and progression. Similarly, histamine H3 receptor (H3R) has long been recognized as a potential therapeutic target for ASD treatment

**Objectives:**

This study aimed to investigate the dual action of A-366, a potent G9a inhibitor with high and selective H3R antagonistic affinity, on the ASD-like behaviours and neuroinflammation of male BTBR T+tf/J mice model.

**Methods:**

Male BTBR T+tf/J mice were housed under standard conditions and treated chronically with A-366 (0.5-2 mg/kg, i.p.) for 21 days. ASD-like behaviors were assessed using the Marble Burying Test, Nestlet Shredding Test, Self-Grooming Test, Spontaneous Alteration Test, Elevated Plus Maze, Light Dark Box, and Three-Chamber Test. Following behavioral testing, cerebellar and hippocampal brain tissues were analyzed using ELISA to quantify pro-inflammatory markers (TNF-α, TNF-β, IL-6, IL-1β, TGF-β) and immunohistochemistry for Iba-1 expression to evaluate neuroinflammation.

**Results:**

A dose dependent decrease of repetitive and anxiety-like behaviours as well as amelioration in social deficits was observed in response to the chronic systemic treatment of A-366 (0.5-2 mg/kg, i.p.). Moreover, A-366 decreased neuroinflammation in cerebellar and hippocampal brain tissues of treated BTBR mice, as evidenced by the reduction in proinflammatory markers TNF-α, TNF-β, IL-6, IL-1β and TGF-β, as well as the decrease in Iba-1 expression.

**Conclusions:**

This in vivo study demonstrates the potential therapeutic value of A-366 as a dual-targeting agent for G9a and H3Rs, with modulatory role on epigenetic, neuroinflammation, and brain histaminergic neurotransmission. Therefore, A-366 is expected to provide a lead template for future design and synthesis of a novel, potent and selective class of drugs to target ASD features.

**Disclosure of Interest:**

None Declared

